# Conserved and acquired: Decoding YbjX and VirK in the pathogenicity of *Shigella flexneri*

**DOI:** 10.1080/21505594.2025.2571677

**Published:** 2025-10-27

**Authors:** Marco Coluccia, Martina Pasqua, Davide Roncarati, Alessandra M. Martorana, Ludovica Altieri, Maria Carmela Bonaccorsi, Alessandra Polissi, Milena Grossi, Bianca Colonna, Gianni Prosseda

**Affiliations:** a Department of Biology and Biotechnology “Charles Darwin”, Pasteur Institute Italy, Sapienza University of Rome, Rome, Italy; b Department of Pharmacy and Biotechnology, University of Bologna, Bologna, Italy; c Department of Pharmacological and Biomolecular Sciences, University of Milan, Milan, Italy; d IBPM Institute of Molecular Biology and Pathology, CNR National Research Council, Rome, Italy; e Department of Biochemical Sciences “Alessandro Rossi Fanelli”, Sapienza University of Rome, Rome, Italy

**Keywords:** E. coli pathogens, virulence, host-pathogen interaction, bacterial envelope, PhoPQ, gene expression

## Abstract

The pathogenicity of *Shigella* spp. human pathogens responsible for bacillary dysentery, results from the combination of factors encoded both on the chromosome and on the virulence plasmid acquired during pathoadaptation. While many key elements have been extensively investigated, several remain poorly characterized. Among these, YbjX and VirK exhibit high structural similarity and are encoded by genes located on the chromosome and the virulence plasmid, respectively. We provide a molecular and functional characterization of *Shigella flexneri* YbjX and VirK. We defined the *ybjX* and *virK* promoter regions and confirmed that their expression is regulated by the PhoPQ two-component system. Localization studies demonstrated that both proteins are cytoplasmic. *In silico* analysis predicted a similar structure for the two proteins, resembling members of the Gcn5-related N-acetyltransferase superfamily. Functionally, lack of VirK resulted in increased permeability of the OM, sensitivity to cationic antimicrobial peptides, and an intensified release of proinflammatory cytokines by infected macrophages (THP-1-derived) and epithelial cells (Caco-2). The deletion of *ybjX* alone didn’t confer detectable phenotypes. When both genes were deleted at the same time a complementary function of YbjX was revealed, as all the phenotypes described above were reinforced. Our findings underscore a synergistic role for YbjX and VirK in OM integrity and the modulation of the host response, suggesting a prominent role of VirK and a supporting role of YbjX. From an evolutionary perspective, our work suggests that the retention of *ybjX* and the acquisition of *virK* reinforced bacterial survival and fitness during the infection of the host.

## GRAPHICAL ABSTRACT

**Figure:**
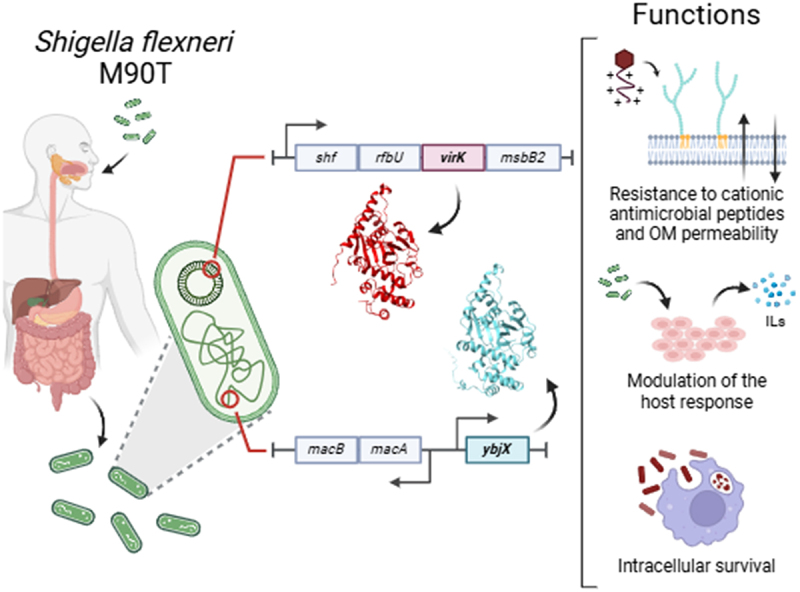

## Introduction

*Shigella* is a highly adapted intracellular human pathogen predominantly found in developing countries, where it causes severe enteric syndromes. Hallmarks of *Shigella* pathogenicity are its capacity to penetrate the colonic epithelium, replicate in and rapidly induce resident macrophage death, enter colonocytes from the basolateral side and spread to adjacent cells through actin-mediated movement [1]. This process ultimately results in the inflammatory destruction of the intestinal barrier. Successful infection requires a fine balance between the pathogen activity and the host response to the infection, *Shigella* finely modulates the host immune responses while simultaneously avoiding host immune surveillance. Therefore, *Shigella* releases factors targeting the pathways required to express genes involved in cell death or encoding antimicrobials and chemoattractants [2,3]. *Shigella* has evolved from its commensal ancestor *Escherichia coli* through convergent evolution involving both the gain and the loss of genes [4,5]. The crucial event toward an intracellular pathogenic lifestyle has been the acquisition of the large virulence plasmid (pINV) which carries the genes for a type III secretion system (T3SS) and its effectors and for intra- and intercellular spreading [6,7]. This process is paralleled by an impressive gene decay of loci which affect the efficient colonization of the host, and of most chromosomal genes useless or redundant for the invasion process [8].

The YbjX protein is encoded by a gene that, although part of the core genome of all bacterial species, belongs to the so-called y-ome, a group of genes with little to no known function [9]. The *ybjX* gene was first discovered during a screening for suppressors of *msbB* in *Salmonella* that fails to myristoylate lipid A within LPS [10]. Functional studies have been limited to two bacterial pathogens, the avian pathogenic *E. coli* (APEC) and *Salmonella enterica*, and provide some suggestions that YbjX contributes to resistance to antimicrobial peptides and the infection ability [11–15]. YbjX has been reported to be homologous to the VirK protein, the prototype of a family of proteins that are involved in the virulence of several bacterial pathogens [14]. The *virK* gene was first identified in *Shigella* and characterized as a pINV gene essential for bacterial spreading among adjacent epithelial cells. *Shigella virK* mutant exhibits a significant decrease in the polymerization of polar F-actin comet tails required for intra- and intercellular movement [16,17]. Subsequently, VirK homologs have been identified in several other bacterial pathogens. In *Salmonella*, *virK* is co-expressed with SPI-2 but, differently from *Shigella*, the gene is encoded on the chromosome. The *Salmonella virK* mutant is less virulent than the wild-type strain and is more susceptible to polymyxin B [14]. The EAEC (enteroaggregative *E. coli*) 042 strain harbors both a chromosomal and a plasmid-borne copy of the *virK* gene with high nucleotide identity [18]. The proposed role for VirK in EAEC is as a component of the chaperone pathways required for the secretion of the plasmid-encoded toxin [19]. A *Shigella* VirK homolog has been found also in *Campylobacter jejuni* and, similarly to *Salmonella*, the deletion of the *virK* gene resulted in an increased sensitivity to antimicrobial peptides and the impairment of the infection in a mouse model [20]. Despite the high gene decay typical of *Shigella* [8,21] and the acquisition of *virK* gene with the pINV, we found that the *ybjX* gene is conserved in the chromosome of all *Shigella* spp. This might point toward a role of YbjX in supporting, possibly together with VirK, the *Shigella* fitness within the host. To date, the *Shigella ybjX* gene is completely uncharacterized both in terms of regulation and function. Concerning *Shigella* VirK, some information is available on its regulation and activity. In the present study, we fully characterize the promoter regions of *ybjX* and *virK* genes, identifying the factors involved in their regulation. Furthermore, we investigate their function, getting more insight into their role in the *Shigella* virulence.

## Materials and methods

### Construction of strains and plasmids

Bacterial strains and plasmids used in this study are listed in Table S1. Oligonucleotides used for chromosomal deletions, protein tagging, plasmid construction, primer extension and qRT-PCR are listed in Table S2. DH10b (EC0113, Thermo Fisher Scientific) was used as the recipient strain in cloning experiments.

Deletion mutant strains were constructed using the one-step method of gene inactivation [22]. M90T Δ*ybjX*, M90T Δ*phoP*, and M90T Δ*phoQ* were obtained by amplifying the kanamycin resistance gene (Km^r^) using pKD4 as a template and the oligo pairs *ybjX*-F/*ybjX*-R, *phoP*-F/*phoP*-R, *phoQ*-F/*phoQ*-R respectively. M90T Δ*virK* strain was obtained using pKD3 as template and *virK*-F/*virK*-R as oligo pair for the amplification of the chloramphenicol (Cm^r^) resistance cassette. Gene deletions together with their antibiotic resistance cassettes were then transduced using P1*vir* phage into M90T wild-type strain to avoid the presence of second-site mutations. P1*vir* transduction was performed according to the protocol described by [23]. Briefly, P1*vir* lysate was prepared by infecting each M90T deletion mutant with P1*vir* phage in LB medium containing 2.5 mM CaCl_2_. The P1*vir* lysate then obtained was used to infect the M90T wild-type strain, and transductants were plated on selective LB agar plates with 10 mM sodium citrate. The M90T Δ*ybjX* Δ*virK* strain was obtained by transferring the Δ*ybjX* (Km^r^) allele into M90T Δ*virK* (Cm^r^) using a P1*vir* phage grown on M90T Δ*ybjX* (Km^r^). The Km^r^ and the Cm^r^ genes in M90T Δ*ybjX* and M90T Δ*virK*, respectively, were removed by introducing the flippase-encoding plasmid pCP20. These antibiotic-sensitive strains were used for complementation assays. The M90T YbjX-His and M90T VirK-His strains expressing His-tagged YbjX and VirK proteins from their native loci (the chromosome for YbjX and the pINV plasmid for VirK) were obtained by amplifying either the *ybjX* or the *virK* gene plus an additional region encoding a 6His tag using the *ybjX*-His-A/*ybjX*-His-B and *virK*-His-A/*virK*-His-B primers pairs. The His-tag was fused to the C-terminus as it is unlikely to interfere with translocation into the periplasm. The Km^r^ cassette was amplified from the pSUB11 plasmid using the primers kan-His-C/*ybjX*-His-D and kan-His-C/*virK*-His-D, respectively. The amplicons obtained were then used as templates to obtain a single larger amplicon with the *ybjX*-His-A/*ybjX*-His-D and *virK*-His-A/*virK*-His-D primer pairs. These PCR products were transformed into a pKD46-containing M90T strain expressing the Red Recombinase. Plasmids pRU*ybjX*, pRU*macA* and pRU*virK* used for GFP reporter assays and primer extension assay were obtained by cloning fragments containing *ybjX, macA* or *virK* regulatory regions, obtained using the pRU*ybjX*-BamHI-R/pRU*ybjX*-BamHI-F, and the pRU*virK*-BamHI-F/pRU*virK*-BamHI-R oligonucleotide pairs, into the BamHI site of the pRU1097 vector upstream of the GFP gene. The region cloned in the pRU*ybjX* and the pRU*macA* plasmids is the same but with opposite orientations. The pRU*ybjX*-MUT plasmid, employed for the analysis of *ybjX* promoter, was obtained by performing site-directed mutagenesis, with pRU*ybjX* as a template and oligonucleotides pRU*ybjX*-MUT-F and pRU*ybjX*-MUT-R. pYbjX and pVirK plasmids used for complementation assays were obtained by cloning a BamHI-restricted fragment containing the *ybjX* (oligonucleotides pYbjX-BamHI-F/pYbjX-BamHI-R) or the *virK* (oligonucleotides pVirK-BamHI-F/pVirK-BamHI-R) genes downstream of Ptac promoter into pGIP7 vector (Table S1). pYbjX and pVirK were transformed in M90T Δ*virK* Cm^S^ and M90T Δ*ybjX* Km^S^, respectively. pYbjX-His plasmid used for the identification of the YbjX translation start codon was constructed by cloning a fragment containing the His-tagged *ybjX* gene and kanamycin resistance gene of M90T YbjX-His into the BamHI site of the pACYC184 plasmid (Table S1). The oligonucleotides employed for the PCR reaction were pRU*ybjX*-BamHI-F/pYbjX-His-BamHI-R. pYbjX-His-M13L, pYbjX-His-M15L and pYbjX-His-M13L-M15L were obtained by site-directed mutagenesis using pYbjX-His as the template, and the oligonucleotides were pYbjX-M13L-F/pYbjX-M13L-R, pYbjX-M15L-F/pYbjX-M15L-R and pYbjX-M13L-M15L-F/pYbjX-M13L-M15L-R, respectively. For PCR screenings and to amplify fragments for cloning, DreamTaq DNA polymerase (EP0702, Thermo Fisher Scientific) and Phusion High-Fidelity DNA polymerase (F-530S, Thermo Fisher Scientific) were employed, respectively. Mutagenesis was performed using the GeneArt site-directed mutagenesis system (A13282, GENEART Invitrogen-Thermo Fisher Scientific). DNA sequencing (Biofab) was utilized to verify the plasmids and confirm the presence of the exact mutations.

### Bacterial growth conditions

Unless stated differently, bacteria were grown aerobically in LB medium at 37°C. 1.6% agar was added to solid media. TSA (Trypticase Solid Agar) was supplemented with 0.01% Congo Red to verify the virulence phenotype of M90T and its derivative strains to be used in cell infections. Antibiotics were used at the following concentrations: ampicillin 50 µg/ml, chloramphenicol 25 µg/ml; gentamycin 10 µg/ml; kanamycin 30 µg/ml; novobiocin 8 µg/ml, polymyxin B 0.625 µg/ml, streptomycin 10 µg/ml. Growth assays were performed by growing M90T and its derivatives in LB broth with the appropriate antibiotic at 37°C. OD_600_ was measured every 30 minutes for up to 10 hours at 37°C in shaking conditions (200 rpm) using ClarioStar (BMG Labtech).

### GFP reporter assay

For the GFP assay, M90T wild-type and its derivatives carrying pRU1097 or pRU1097-derived plasmids (Table S1) were grown in M9 complete medium with 10 µg/ml gentamycin at 37°C until the exponential phase (OD_600_ ~ 0.6). Cultures were diluted again to OD_600_ 0.2 in a final volume of 200 µL in each well of a 96-well plate. The plates were incubated for 1 hour at 37°C in shaking conditions (200 rpm), and then both OD_600_ and GFP fluorescence intensity were measured using a ClarioStar (BMG Labtech). Fluorescence values were divided by the absorbance at 600 nm to normalize to bacterial cell concentration and the background signal was subtracted.

### Primer extension assay

Total RNA from exponentially growing *E. coli* DH10b cells, carrying plasmids pRU*macA*, PRU*ybjX* or pRU*virK*, was extracted with TRI-Reagent (15596026, Thermo Fisher Scientific), according to manufacturer’s protocol. Primer extension analysis was performed as previously described [24], with some modifications. 15–20 µg of total RNA were precipitated and resuspended in 10 μl of nuclease-free milli-Q water, containing 0.1 pmol of 5’-^32^P-labeled specific primers (*macA*-PE3 for P*macA*; *ybjX*-PE3 for P*ybjX*; shf-PE2 for Pshf, see Table S2). Following RNA denaturation for 5 minutes at 65°C, 4 μl of 5X Reaction buffer, dNTPs (1 mM final concentration) and 100 U of RevertAid Reverse Transcriptase (K1691, Thermo Fisher Scientific) were added to each reaction and reverse transcription was carried at 42°C for 60 minutes. Samples were incubated for 10 minutes at 25°C with 10 μg of RNase A, extracted with phenol-chloroform, ethanol precipitated, and resuspended in 10 μl of Formamide Loading Buffer (95% formamide; 10 mM EDTA; 0.01% Xylene Cyanol and Bromophenol Blue). Samples were then analyzed on a denaturing 6% polyacrylamide gel in parallel with sequencing reaction products obtained using the same primers. At the end of the electrophoretic separation, the gel was blotted onto a 3 MM Whatman paper sheet, dried and the radioactive bands were visualized using a phosphorimager. For promoter predictions, the BPROM tool was used [25] after experimentally identifying the transcription start site. When no promoter could be identified using BPROM, −10 and −35 sequences were determined manually.

### Immunoblotting and protein localization

Bacterial cultures were grown overnight in LB medium at 37°C in shaking conditions, then cultures were diluted 1:100 and grown until OD_600_ ≈0.6. Samples were prepared by suspending bacterial pellets in PBS (Phosphate-Buffered Saline) with 1x Laemmli buffer, normalized to cell density, boiled at 100°C and loaded in 12.5% SDS-PAGE gels. Proteins were transferred on a nitrocellulose membrane (GE10600124, Amersham, Sigma Aldrich) and blocking was carried out for 1 hour with 5% dry skimmed milk in TBS-T (TBS with 0.1% Tween20). Incubation with primary anti-His monoclonal antibodies (H1029, Sigma-Aldrich) and anti-GroEL mouse monoclonal antibodies (ADI-SPS-870-F, Enzo) was performed overnight at 4°C in TBS-T containing 2.5% dry skimmed milk. We used horseradish peroxidase (HRP)-conjugated goat anti-mouse secondary antibodies (S372B, Promega). Membranes were washed in TBS-T and developed in ECL (3577, Thermo Fisher Scientific). Pictures were acquired using a ChemiDoc system (BioRad) and the relative amount of protein was quantified using the ImageLab software (BioRad). To determine YbjX and VirK subcellular localization, overnight cultures of *S. flexneri* M90T expressing either *virK-his* (M90T VirK-His) or *ybjX-his* (M90T YbjX-His) (Table S1) genes were diluted 1:100 in fresh medium and grown to the mid-logarithmic phase. Periplasmic, cytoplasmic, inner (IM) and outer (OM) membrane fractions were prepared as described previously [26], with some modifications. Spheroplasts were lysed by a single cycle through a Cell Disrupter (One Shot Model, Constant Systems Ltd.) at 21 kPsi. Protein concentrations were measured by Bradford protein assay (5000002, BioRad) and equal amounts of proteins from each fraction were analyzed on a 12.5% SDS-PAGE gel. YbjX and VirK were detected by immunoblotting using the anti-His monoclonal antibodies (H1029, Sigma-Aldrich). For confirmation of good fractionation, the anti-LptC [27] was used as a marker of the IM fraction, the anti-DegP (CSB-PA314631ZA01ENV, CusaBio) and anti-OmpF (CSB-PA365808ZA01ENV, CusaBio) were used as markers of periplasmic and the OM fractions, respectively, and the anti-GroEL (ADI-SPS-870-F, Enzo) was used as marker of the cytoplasm.

### Cell culture and infection

For infection experiments, THP-1 cells (TIB-202, American Type Culture Collection) and Caco-2 cells (HTB-37, American Type Culture Collection) were used. Cells were grown at 37°C and 5% CO_2_ humidified atmosphere as described previously [28,29]. The medium used for THP-1 was RPMI 1640 (31870–074, Gibco, Thermo Fisher Scientific) containing 10% heat-inactivated fetal bovine serum (FBS) (26140079, Gibco, Thermo Fisher Scientific), 2 mM L-glutamine and PS (0.05 I.U./mL penicillin and 0.05 I.U./mL streptomycin), referred to as RF10; for Caco-2 cells, the medium was DMEM (41966–052, Gibco, Thermo Fisher Scientific) containing 10% FBS and PS, referred to as DF10. THP-1 cells were seeded in 35 mm plates at a density of 1x10^6^ cells/plate in RF10 supplemented with 50 nM PMA for differentiation into macrophages. After 48 hours, RF10 with PMA was replaced with fresh RF10, and two hours before infection medium was replaced with RPMI. Caco-2 cells were plated in 35 mm plates at a density of 0.8x10^6^ cells/plate in DF10. After 24 hours, DF10 was removed and cells were starved with DMEM supplemented with 0.5% FBS and PS (DF0.5). Two hours before the infection, DF0.5 was replaced with DMEM. The same protocol described above for Caco-2 cells was used to grow and seed human embryonic kidney cells HEK-293 (CRL-1573, American Type Culture Collection). Infection experiments with M90T and its derivative strains were performed at MOI 100. Infected cells were centrifuged (15 minutes, 750 g) and incubated for 30 or 45 minutes (THP-1 and Caco-2/HEK-293, respectively) at 37°C in a 5% CO_2_ atmosphere. Plates were washed three times with 1x PBS to remove extracellular bacteria (time zero T0). To kill extracellular bacteria, RPMI for THP-1 cells or DMEM for Caco-2/HEK-293 cells supplemented with gentamicin (100 µg/ml) was added. Plates were incubated at 37°C up to 3 (THP-1) or 4 h (Caco-2 and HEK-293).

Live and dead assay was performed by staining intracellular bacteria with DAPI, which stains the entire bacterial population, and propidium iodide (PI), to selectively stain dead bacteria due to their increased membrane permeability, as previously described [30].

The IL-1β secreted by infected THP-1 cells was measured in the supernatant harvested 2 hours post-infection by using the Human IL-1 beta/IL-1F2 DuoSet ELISA kit (DY201, R&D Systems). The release of IL-8 by infected Caco-2 and HEK-293 cells was evaluated in the supernatant collected 4 hours post-infection using the Human IL-8 ELISA kit (EH2IL8, Thermo Scientific).

### LDH cytotoxicity assay

CyQUANT LDH Cytotoxicity Assay kit (C20301, Invitrogen, Thermo Fisher Scientific) was used to measure THP-1 cytotoxicity induced by M90T and its derivatives as previously described [28]. Supernatants were harvested at different time points post-infection and LDH activity was determined by measuring absorbance at 490 nm and 680 with a CLARIOstar plate reader (BMG Labtech). The percentage of cytotoxicity was calculated as indicated by the manufacturer as the ratio of the LDH activity of the supernatant of infected cells minus the spontaneous LDH activity and the maximum LDH activity minus the spontaneous LDH activity.

### RNA isolation and real-time PCR

Bacterial RNA purification from intracellular bacteria was performed as previously described [31], and cDNA synthesis was achieved with the high-capacity cDNA reverse transcription kit (4368814, Applied Biosystems, Thermo Fisher Scientific). 1 µg of total bacterial RNA was treated with DNase I and then retrotranscribed in a 20 µL-reaction mix following the manufacturer’s instruction. Quantitative reverse transcription PCR was performed on StepOne real-time PCR system (4309155, Applied Biosystems, Thermo Fisher Scientific). The reaction volume was 20 µL, with Power SYBR green PCR master mix (Applied Biosystems, Thermo Fisher Scientific), 2 µL of DNA samples, and 300 nM of oligonucleotides for *ybjX*, *virK*, and *nusA* genes. Cycle conditions were as follows: 1 cycle at 95°C for 2 minutes, 40 cycles at 95°C for 10 seconds, and 60°C for 30 seconds. Relative quantification was performed using the comparative cycle threshold 2^−ΔΔCT^ method. All qRT-PCR primer pairs designed using Primer Express v2.0 are shown in Table S2 and validated experimentally.

### Plaque assay

Caco-2 cells were seeded in 60 mm plates at 5x10^6^ cells/plate density in DF10. After 24 hours, cells were serum starved in DF0.5. Two hours before bacterial infection, DF0.5 was replaced with fresh DMEM, containing only L-glutamine. The infection was carried out with an MOI of 10^−3^. Plates were centrifuged at 750 g for 15 minutes and then incubated at 37°C under a 5% CO_2_ humidified atmosphere for 45 minutes to enhance bacterial entry into epithelial cells. Extracellular bacteria were removed by extensive PBS washes, and the infected monolayer was overlayed with 0.5% agarose in DMEM containing 100 µg/ml gentamycin and 0.5% FBS. After 72 hours the agarose overlayer was removed from every plate, cells were fixed with 95% ethanol and stained with 5% Giemsa solution for plaque counting. Pictures of the single plaques were acquired using a ZOE Cell Imaging System (BioRad), to evaluate the area with ImageJ.

### Fluorescence microscopy and image acquisition

Caco-2 cells were seeded on coverslips in 35 mm plates at 0.8x10^6^ cells/plate density in DF10 and the infection was performed as described above. Four hours post-infection cells were washed with PBS, fixed with 4% paraformaldehyde (20 minutes) and permeabilised with 0.5% TritonX-100 (10 minutes). Incubations with TRITC-labeled phalloidin (P1951, Sigma-Aldrich) (30 minutes) and 0.5 μg/ml 4,6-diamino-2-phenylindole (DAPI, D9542, Sigma-Aldrich) (3 minutes) were performed at room temperature. Images were acquired at the IBPM imaging platform with a Nikon Ti2 confocal spinning disk microscope (implemented with Crest X-Light V3 module from CrestOptics) equipped with the Kinetix sCMOS camera (Teledyne Photometrics), a 60× (immersion oil, NA 1.4) objective and CELESTA lasers (Lumencor). Image acquisition and analyses were performed using NIS-Elements AR software modules (Nikon). Image projections from z-stacks were created using the Maximum Intensity Projection (MIP) function.

### Statistical analysis

Statistically significant differences were identified using a two-tailed Student’s *t*-test or one-way ANOVA with Tamhane’s T2 post-hoc test for multiple comparisons. p-values are indicated in the supplementary file “Statistical analysis.” All experiments were replicated at least 3 times with independent biological replicates and, additionally, when multiwell plates were used (e.g. fluorescence and absorbance measures, RT-qPCR) every sample was measured three times in different wells (technical replicate).

## Results

### Molecular characterization of the *ybjX* and *virK* promoters

**Figure 1.**
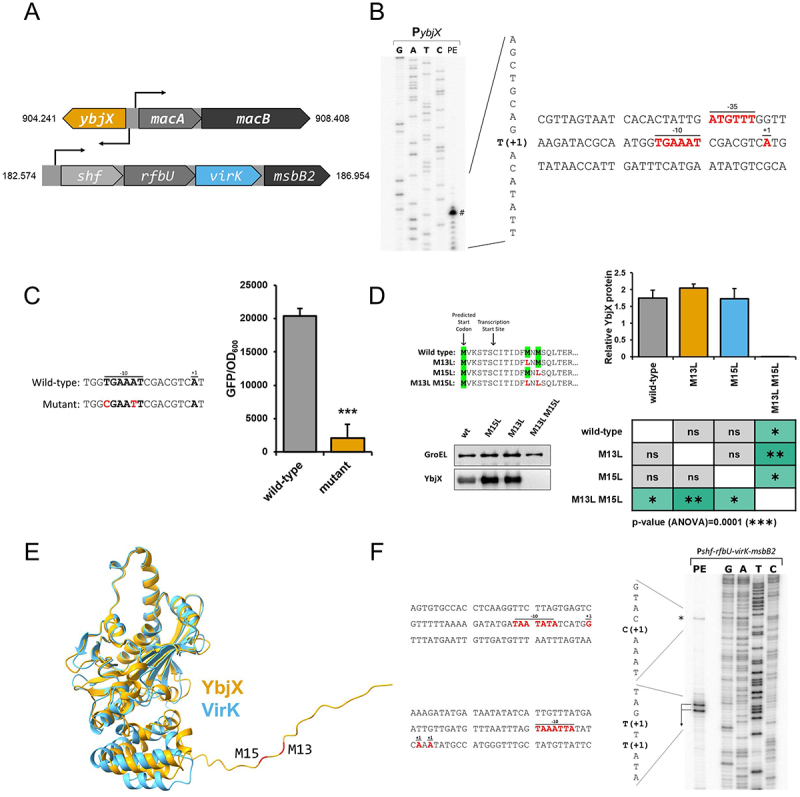
Characterization of *ybjX* and *virK* genetic loci. (A) Representation of the *ybjX-macAB* locus and the *shf-rfbU-virK-msbB2* operon, on the chromosome and the pINV, respectively. (B) Identification of the *ybjX* promoter. The 5’ end of the *ybjX* transcript was identified through primer extension analysis. On the right, a summary of the relevant features within the nucleotide sequence of the *ybjX* promoter region is presented. The transcriptional start site (+1) and the −10 and −35 regions are shown in red. (C) Activity of *ybjX* promoter shown in (B). *Left*: two site-specific mutations were introduced at positions −9 and −13 (in red) in the −10 region of the *ybjX* promoter. *Right*: transcriptional activity of the wild-type (TGAAAT) and mutated (CGAATT) *ybjX* promoter was evaluated by assessing the expression of the GFP reporter gene cloned downstream. Values represent the ratio between the fluorimetric units and the optical density at 600 nm. (D) Determination of the coding sequence of *ybjX*. The *ybjX* gene carrying a His tag was cloned into the pACYC184 vector and the codons for two possible methionines at position 13 and 15 were mutated from ATG to CTG and YbjX protein production was evaluated. *Left*: a representative Western blot is shown. *Right*: densitometric analysis of Western blots from three independent experiments. (E) The superimposition of the AlphaFold-predicted structures of *S. flexneri* YbjX (in yellow) and VirK (in light blue) shows their structural homology and the presence of an unstructured chain at the N-terminal of YbjX. (F) Identification of the *virK* promoter. The 5’ end of the *virK* transcript was identified through primer extension analysis. On the left, a summary of the relevant features within the nucleotide sequence of the *virK* promoter region is presented. The transcriptional start site (+1) and the −10 and −35 regions are shown in red. Statistical significance was determined using a two-tailed Student’s t-test for Figure 1C and using a one-way ANOVA test with Tamhane’s T2 post-hoc test for Figure 1D. *0.05≥*p* > 0.01; ** 0.01≥*p* > 0.001: *** 0.001≥*p* > 0.0001. Error bars represent SD.

A first survey on the *Shigella* genome indicates that both *ybjX* and *virK* genes are well conserved across the four subspecies of *Shigella*. Hereafter *Shigella* refers to the *S. flexneri* 5a M90T strain [7], which was utilized in all experiments described below. The *ybjX* gene is part of the *ybjX-macAB* locus in the chromosome and is divergently transcribed from the *macAB* operon encoding an MDR efflux pump (Figure 1A). The *virK* gene is located in the pINV *shf-rfbU-virK-msbB2* operon (Figure 1A), whose genes are mainly involved in the biosynthesis of the components of the outer membrane. In both cases, the promoter regions have not been experimentally defined. A primer extension analysis of the transcripts generated under the control of the P_*macAB*_ and P_*ybjX*_ promoters allows us to identify the *macA* transcription start site, an A positioned 73 bp upstream of the ATG of *macA*, and the associated −10 and −35 promoter sequences boxes (Fig S1A). Surprisingly we identified the transcription start site of *ybjX* as a T positioned 18 bp downstream of the predicted ATG (Figure 1B) [32]. We introduced two site-specific mutations in the −10 region of the predicted *ybjX* promoter, previously cloned upstream of the GFP gene (pRU*ybjX* Table S1). The substitution of A with T and T with C at position −9 and −13, respectively, completely abolishes the expression of the GFP gene, confirming the identification of the actual promoter (Figure 1(C)). Overall, these data allow us to define the transcription start sites present in this regulatory region and to observe that between the two transcription start sites, there is only a region of 58 base pairs containing the two −35 regions that are partially overlapping (Fig S1B). The predicted sequence of the YbjX protein nicely aligns (34.4% identity) with that of the *Shigella* VirK protein but shows an extended N-terminal region of over 20 amino acids. Considering that the transcription start site of *ybjX* is located downstream of the previously annotated start codon of the coding sequence [32], we decided to experimentally determine the YbjX ATG start codon. Close examination of the *ybjX* annotated gene showed two methionine codons located at positions 13 and 15 after the predicted translation start codon (Figure 1(D)). To define the initiation codon, site-specific mutagenesis was carried out on a his-tagged *ybjX* gene (pYbjX-His Table S1), introducing mutations from ATG to CTG at either single methionine codons or both. YbjX protein production was then evaluated by Western blot analysis of *E. coli* extracts carrying the different constructs (pYbjX-His-M13L; pYbjX-His-M15L and pYbjX-His-M13L-M15L, Table S1) (Figure 1(D)). While we did not observe differences in the YbjX level between the strain containing wild-type sequence and those containing the mutation in the single methionine codons, mutation of both ATG completely abolished YbjX translation. This suggests that the translation of YbjX can start equally from one of the two codons, probably due to their proximity. The AlphaFold structural models of YbjX, based on the longer predicted amino acid sequence, and VirK predict a superimposable structural organization for the two proteins (Figure 1E). The experimentally-defined translation start sites of YbjX are located downstream of the predicted one and cause only the shortening of the unstructured N-terminal chain without affecting the three-dimensional structure of the protein. Moreover, the two translation start codons are only one residue apart and are located in the unstructured N-terminal chain, suggesting that the two protein isoforms do not significantly differ neither structurally nor functionally. As for *virK*, the position of the promoter of the *shf-rfbU-virK-msbB2* plasmid operon has been only suggested by the presence of PhoP-binding sites, which usually overlap with the −35 sequence [33], but experimental data to clearly define the TSS are currently lacking. A primer extension assay using pRU*virK* indicates that the transcript containing *virK* has two alternative 5’ sites separated by a single nucleotide, as evidenced by the presence of two distinct bands. These two sites are only 1 bp distant from each other and are located about 100 bp from the *shf* coding sequence, preceded by a −10 sequence TAAATT (Figure 1F). The primer extension assay revealed also the presence of a third potential site upstream with a faint band, suggesting that transcription may also start from there with lower efficiency.

### *ybjX* expression is activated by the PhoPQ two-component system

**Figure 2.**
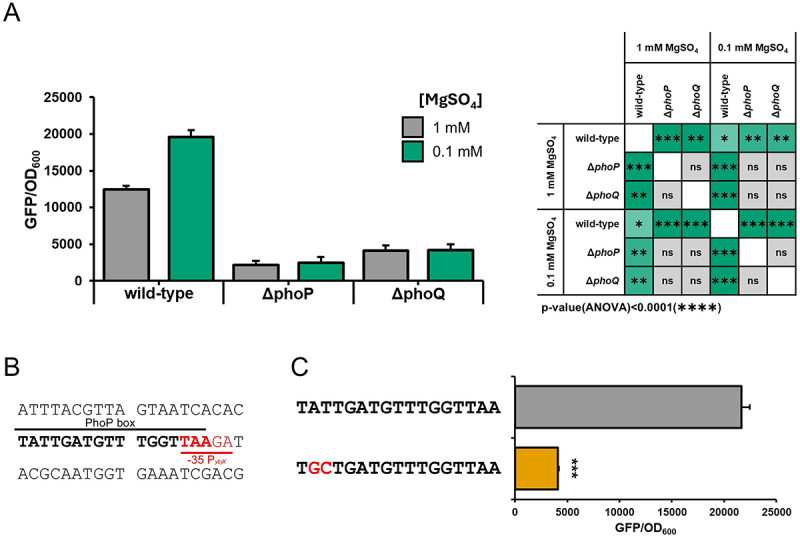
*ybjX* is among the genes under the control of the PhoPQ TCS. (A) The transcription of *ybjX*, measured by evaluating the expression of the GFP reporter gene cloned downstream of the P_*ybjX*_ in the pRU*ybjX* vector, is enhanced by low concentrations of magnesium (0.1 mM MgSO_4_ compared to 1 mM MgSO_4_) in M9 minimal medium, but is hampered by the deletion of the *phoP* and *phoQ* genes. (B) Identification of the TATTGA-5bp-GGTTAA PhoP box (boldface) overlapping the −35 region of the P*ybjX* (red). (C) Validation of the PhoP box. AT at − 39 and −40 were mutated to GC in the pRU*ybjX* plasmid. Bacteria were grown in M9 containing 0.1 mM MgSO_4_ and the promoter activity was evaluated by assessing the GFP expression. Values represent the ratio between the fluorimetric units and the optical density at 600 nm. Statistical significance was determined using a one-way ANOVA test with Tamhane’s T2 post-hoc test for Figure 2A and a two-tailed Student’s t-test for Figure 2C. *0.05≥*p* > 0.01; ** 0.01≥*p* > 0.001; *** 0.001≥*p* > 0.0001. Error bars represent SD.

Pathogenic bacteria often use two-component systems (TCSs) to modulate gene expression in response to the various niches encountered during host infection. In several *Enterobacteriaceae*, including *Shigella*, it has been demonstrated that the PhoPQ system contributes to coordinate the expression of the virulence phenotype in response to different stimuli [34,35]. In this context, the response regulator PhoP has been reported to control the expression of the *ybjX* gene in *S. enterica* [14], and the *shf-rfbU-virK-msbB2* operon in *Shigella* [33,36]. Given that, we investigated whether PhoP also regulates the expression of the *ybjX* gene in *Shigella*. We observed that *ybjX* expression, monitored through transcriptional fusions with the GFP reporter gene (pRU*ybjX* Table S1), is enhanced in the presence of a low concentration of Mg^2+^ (0.1 mM) (Figure 2A), a typical signal sensed by the PhoPQ TCS. Moreover, we found that the absence of either the regulator PhoP or the sensor PhoQ is associated with a significant reduction of GFP fluorescence independently of the concentration of Mg^2+^ (Figure 2A), suggesting that *ybjX* expression is positively regulated by the PhoPQ system. Several studies indicate that PhoP mainly binds to the (T/G)GTTTA-5bp-(T/G)GTTTA consensus sequence, even if many variations have been reported [37]. By analyzing the regulatory region of *ybjX* using the PePPER tool [38] for the prediction of transcription factor binding sites, we found a potential PhoP box (TATTGA-5bp-GGTTAA) overlapping the −35 promoter sequence (Figure 2B). We substituted AT with GC (at −39 and −40) and measured the transcriptional activity of the *ybjX* promoter in PhoP-inducing conditions (0.1 mM Mg^2+^). The mutation is located in the distal half of the PhoP box and was chosen because this position is among those whose importance for PhoP binding has been confirmed for the transcription activation of other PhoP-regulated genes [39]. The alteration of the PhoP box was associated with a significant reduction of *ybjX* expression, suggesting that the transcription of *ybjX* is under the direct control of PhoP (Figure 2C). Besides sharing structural homology, YbjX and VirK are also regulated by the PhoPQ two-component system. This shared regulation strongly suggests that both proteins play a role in responding to similar environmental or physiological conditions, potentially contributing to related biological processes or stress responses.

### *YbjX* and *VirK* are cytoplasmic proteins important for the outer membrane permeability

**Figure 3.**
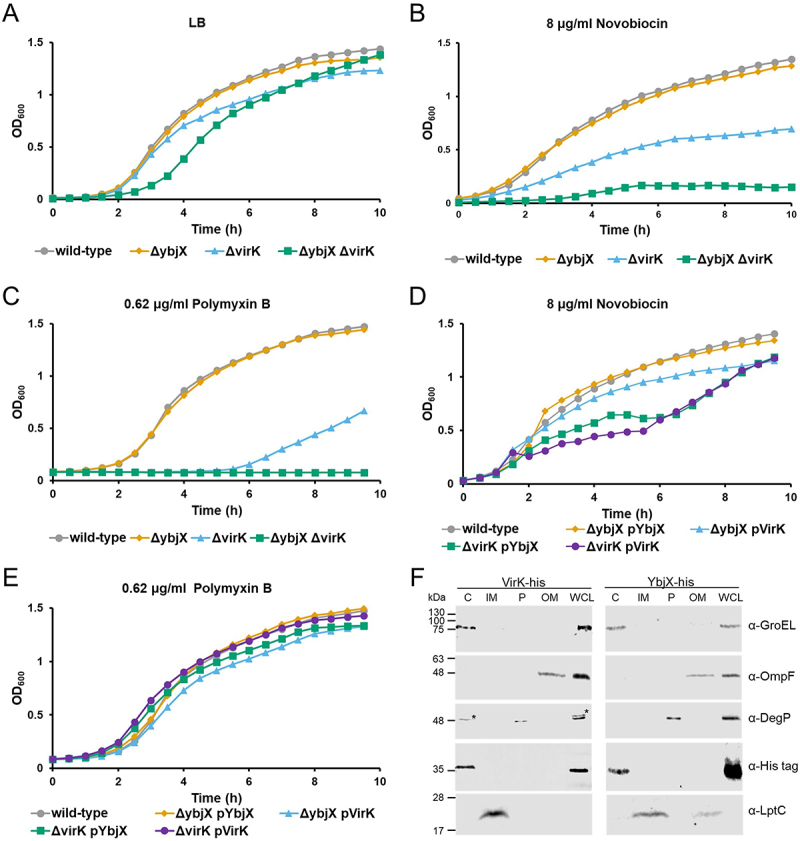
YbjX and VirK are cytoplasmic proteins involved in membrane permeability. (A, B, C, D and E) Growth of *S. flexneri* M90T wild-type and its derivatives in LB medium and in the presence of novobiocin (8 μg/ml) and polymyxin B (0.62 μg/ml). Bacterial growth was monitored in LB medium for up to 10 hours. The growth curves shown derive from one of three experiments, which gave similar results. (F) Subcellular localisation of VirK-His and YbjX-His proteins. Whole cell lysate (WCL) and cytoplasmic (C), inner membrane (IM), periplasmic (P), and outer membrane (OM) fractions from a *S. flexneri* M90T expressing either *virK*-His or *ybjX*-His were prepared and analysed by Western blotting using monoclonal anti-His antibodies. The anti-DegP and anti-GroEL antibodies were used as markers for periplasmic and cytoplasmic fractions, respectively, whereas the anti-LptC and anti-OmpF antibodies were used as markers for IM and OM fractions, respectively.

Taking into account that many of the genes that constitute the PhoP regulon are involved in the remodeling of the cell envelope, including lipid A modification and changes in the OM protein composition [40], a contribution of YbjX and VirK to *Shigella* OM integrity can be envisaged. Before investigating the permeability of the OM of various M90T strains harboring the deletion of *ybjX* and/or *virK*, we evaluated the growth in LB medium (Figure 3A), showing that all strains have similar growth properties, despite a slightly extended lag phase for the Δ*ybjX*Δ*virK* strain. OM permeability properties of the different strains were examined by exposing them to novobiocin, a small molecule to which OM is naturally impermeable [41]. Following the growth curve of the M90T derivatives in the presence of 8 µg/ml of novobiocin, we found that, while M90T Δ*ybjX* behaves like the wild-type, the growth of M90T Δ*virK* is significantly impaired (Figure 3B). Interestingly, although *ybjX* seems to be dispensable in the wild-type *Shigella*, its deletion largely increases the sensitivity of M90T Δ*virK* to the antibiotic. Indeed, the presence of novobiocin in the medium almost completely inhibited the growth of M90T Δ*ybjX*Δ*virK* (Figure 3B). These findings indicate that VirK exerts a dominant function for the *Shigella* OM integrity and suggest a role also for YbjX in controlling OM permeability, as its presence attenuates the phenotype conferred by the lack of VirK. It has been shown that in *S. enterica* the deletion of *ybjX* or *virK* increases the bacterial sensitivity to the antibiotic polymyxin B [14], a cationic peptide whose positively charged and hydrophobic regions can interact with the LPS forming a crystalline structure with subsequent membrane disruption [42]. The sensitivity of the M90T deletion mutants to 0.62 µg/ml of polymyxin B was assessed by following the bacterial growth curves in the LB medium. The growth properties of the *ybjX* mutant were not affected by the presence of the antibiotic, while the lack of VirK made M90T highly sensitive to polymyxin B compared to the wild-type strain (Figure 3C). Similar to what was observed in the novobiocin experiments, the phenotype conferred by *virK* deletion was further exacerbated in the Δ*ybjX*Δ*virK* double mutant, which was the most sensitive to polymyxin B (Figure 3C). To verify whether the structural homology of YbjX and VirK proteins translated into a functional homology, the M90T Δ*ybjX* and M90TΔ*virK* single mutants were cross-complemented by overexpressing VirK and YbjX, respectively. Additionally, we also confirmed the results discussed above for growth in the presence of novobiocin and polymyxin B by measuring growth of complemented strains (Figure 3D,E). This complementation and cross-complementation approach was chosen to focus on the study of the functional homology of the two proteins, removing external factors that may influence the expression of the *ybjX* and *virK* genes in their native loci. Interestingly, overexpression of YbjX can functionally cross-complement the lack of VirK, restoring a resistance level similar to that displayed by the wild-type strain (Figure 3D,E). Altogether, the results obtained from OM permeability and polymyxin B resistance experiments point to a relevant function of YbjX and VirK that contribute to OM integrity/remodeling. Concerning the cell localization of these proteins, there are contradictory results on VirK which was found to be a cytoplasmic protein in *C. jejuni* [20] and localized in the periplasm in EAEC [19], while Song and coworkers regarded YbjX as OM protein [11,12] without experimental evidence. The amino acid sequence analysis of YbjX and VirK did not evidence any signal sequence supporting its export across the inner membrane (IM). To define where YbjX and VirK localize in *Shigella*, we conducted cell fractionation experiments. Cell fractions of M90T expressing YbjX-His or VirK-His were prepared as described in Materials and Methods and the absence of cross-contamination between them was assessed by Western blot using specific proteins as markers. Probing of the filters with a monoclonal anti-His antibody revealed that both YbjX and VirK reside in the *Shigella* cytoplasm (Figure 3F). As for the protein structure, the AlphaFold structural models of YbjX and VirK predict a similar structural organization with a three-layer αβα sandwich fold with a central beta-sheet with five antiparallel strands and a short sixth parallel strand (Fig S2). A short N-terminal domain (about 70 residues) and a larger C-terminal domain (about 250 residues) can be observed in both structural models, with a positively charged region between the two domains (Fig S2). Analysis with VAST (Vector Alignment Search Tool at NCBI) identified among proteins that share the three-layer αβα sandwich architecture of the large domain, many proteins that belong to the Gcn5-related N-acetyltransferase (GNAT) superfamily. Intriguingly, the small domain appears to bear structural similarity to a CoA-transferase protein (among others). Thus, it is tempting to speculate that YbjX and VirK may participate in the OM biogenesis pathways functioning as modulators of proteins involved in OM remodeling.

### Expression of *virK* and *ybjX* increases upon host cell infection

**Figure 4.**
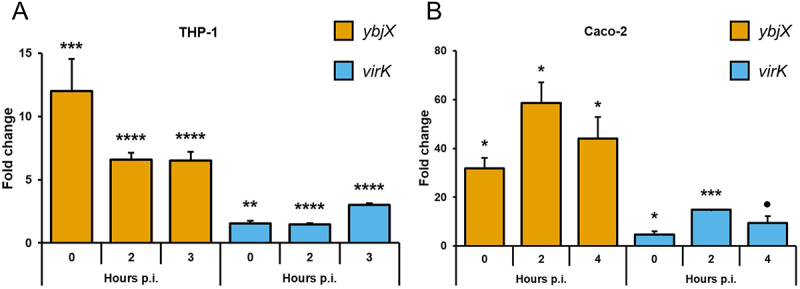
Modulation of *ybjX* and *virK* expression during *S. flexneri* M90T intracellular life inside THP-1-derived macrophages (A) and Caco-2 epithelial cells (B). The quantitative analysis of *ybjX* and *virK* expression was performed utilizing a real-time qPCR assay. Total RNA was extracted from intracellular bacteria at the indicated time points p.i. 0 h corresponds to bacterial adhesion to the target cells (see materials and methods). The y-axis indicates the expression fold-change (RQ value) for each gene. All infection experiments were repeated three times and at least three wells were run for each sample. Statistical significance was determined using a one-way ANOVA test with Tamhane’s T2 post-hoc test. ●0.1≥*p* > 0.05; *0.05≥*p* > 0.01; **0.01≥*p* > 0.001; ***0.001≥*p* > 0.0001; ****p ≤ 0.0001. Error bars represent SD.

Based on the role of YbjX and VirK role in OM permeability/modifications, we asked whether these proteins may play relevant functions during *Shigella* infection. For this, we first investigated whether the expression of *ybjX* and *virK* is modulated upon *Shigella* invasion of THP-1-derived macrophages and Caco-2 epithelial cells. Figure 4 shows that transcription of both genes is activated in intracellular bacteria, with *ybjX* being the most upregulated. Depending on the infected cell type, we however observed differences in the level and kinetics of activation. In *Shigella* infecting THP-1-derived macrophages the highest upregulation (more than 10-fold) of *ybjX* was seen at T0, as soon as bacteria entered the cells, while the activation peak of *virK* was reached 3 hours post-infection (p.i.) (Figure 4A). The activation levels of *ybjX* and *virK* in Caco-2 epithelial cells were significantly higher than in THP-1 cells and both genes exhibited similar kinetics, peaking at 2 hours post-infection (≥50-fold and 10-fold for *ybjX* and *virK*, respectively) (Figure 4B).

### YbjX and VirK activity influences the release of IL-8 by infected Caco-2 cells

**Figure 5.**
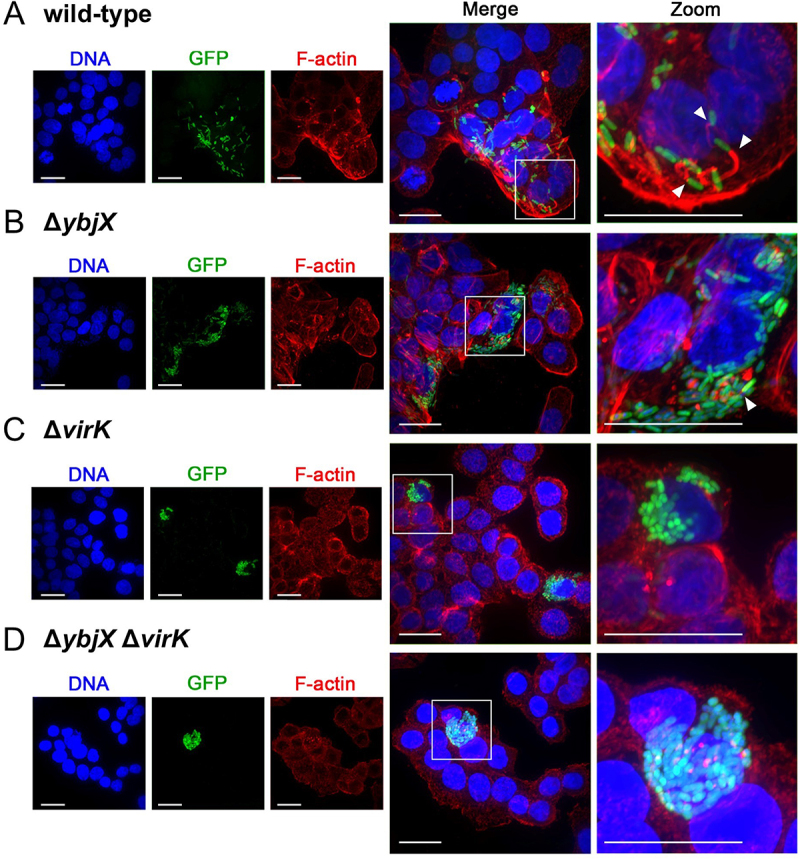
The deletion of *virK* affects *S. flexneri* M90T ability to spread intercellularly. Caco-2 cells were infected with M90T wild-type (A) or its Δ*ybjX* (B), Δ*virK* (C) and Δ*ybjX*Δ*virK* (D) derivatives harboring the pGEM-GFP vector (green). Samples were fixed 4 hours post-infection with 4% paraformaldehyde, permeabilised and incubated with phalloidin-TRITC (red) and with 4,’6-diamino-2-phenylindole (DAPI, blue) to visualize F-actin and DNA, respectively. Scale bar, 20 µm. Merged panels show a 1.8x magnification with respect to the single channels. The rightmost column shows a zoom-in (1.8x) of the framed region. White arrowheads indicate the polymerization of F-actin, which cannot be observed in the cells challenged with the *S. flexneri* M90T Δ*virK* and Δ*ybjX*Δ*virK* strains.

**Figure 6.**
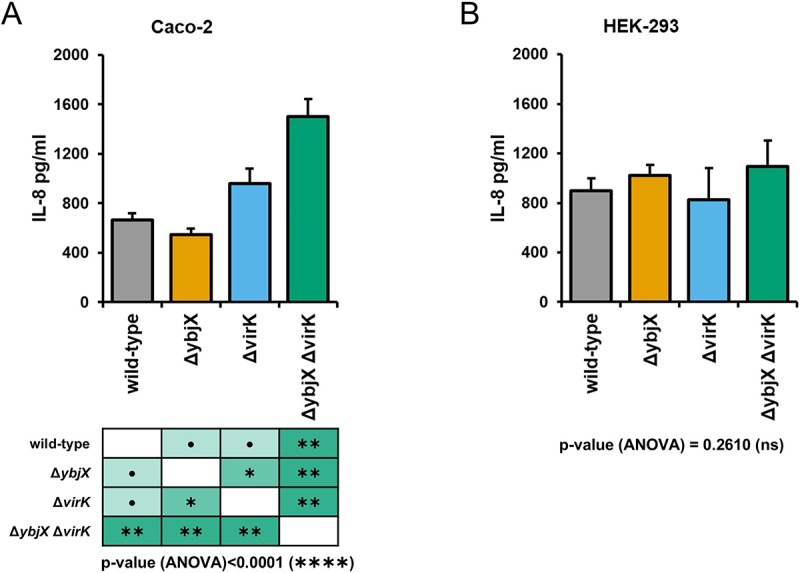
IL-8 release by Caco-2 (A) and HEK-293 (B) cells infected with *S. flexneri* M90T and its derivatives. Supernatants from infected cells were recovered 4 hours post-infection and IL-8 concentration was determined by ELISA assay. All infection experiments were repeated three times and at least three wells were run for each sample. Statistical significance was determined using a one-way ANOVA test with Tamhane’s T2 post-hoc test. ●0.1≥*p* > 0.05; *0.05≥*p* > 0.01; **0.01≥*p* > 0.001. Error bars represent SD.

The *ybjX* and *virK* expression profiles observed in intracellular bacteria suggest that these proteins may play a significant role during *Shigella* infection. As for VirK, it is long known that it is involved in *Shigella* cell-to-cell spreading into the epithelial monolayer by indirectly influencing post-translational modifications of IcsA [17]. By contrast, the function of *Shigella* YbjX in epithelial cell invasion has never been explored. Based on the observation that VirK and YbjX show structural and functional homology, and are capable of cross-complementation for OM integrity, we asked whether YbjX has any role in *Shigella* intercellular spreading. To this purpose, we carried out plaque assay experiments by infecting epithelial Caco-2 cell monolayers with *S. flexneri* M90T wild-type or derivative strains where the *ybjX* gene, the *virK* gene or both were deleted. Seventy-two hours p.i. the Caco-2 cell monolayers were observed for the presence of plaques. Results show that the wild-type and the *ybjX* mutant strains formed a comparable number of plaques (Fig S3A) with similar size (Fig S3B), while the *virK* mutant, as the double mutant, could not give rise to any plaque (Fig S3A). Accordingly, Figure 5 and Fig S3C show that, while the wild-type M90T strain and the Δ*ybjX* derivative form F-actin tails and spread into the Caco-2 monolayer, both Δ*virK* and Δ*ybjX*Δ*virK* efficiently multiply inside the entry cell but remain confined therein, being unable to spread to adjacent cells and to polymerize actin tails. Parallelly, Caco-2 cell monolayers were infected with M90T *virK* and *ybjX* deletion mutants complemented and cross-complemented with pYbjX and pVirK. Results from plaque assay showed that, although overexpressed, YbjX was unable to recover, not even partially, the spreading ability of M90T Δ*virK* (Fig S3A). Interestingly, overexpression of VirK in M90T Δ*ybjX* gives rise to larger plaques compared to the wild-type or to the Δ*virK* pVirK strains (Fig S3B). Overall, these data suggest that the activity of VirK regarding *Shigella* cell-to-cell movement is unique and cannot be extended to YbjX, despite the high sequence/structural homology. However, as reported above, we found that both proteins are relevant for OM permeability, implying their involvement in OM modification/integrity. Banking on the knowledge that the OM stability/modifications have a main role in evoking the cellular innate response to bacterial invasion, we wondered whether the YbjX and VirK activity might impact the release of Interleukine-8 (IL-8) by epithelial cells. For this, the concentration of IL-8 in the supernatants of Caco-2 and HEK-293 cells infected with M90T wild-type, Δ*ybjX*, Δ*virK* or Δ*ybjX*Δ*virK* strains, was evaluated by ELISA 4 hours p.i. As shown in Figure 6A, infection with M90T Δ*ybjX* did not significantly alter the level of IL-8 released by Caco-2 cells compared to wild-type M90T cells. By contrast, M90T lacking the *virK* gene increased the concentration of IL-8 in the supernatant from infected Caco-2 by more than 40% (Figure 6A). Interestingly, infection with M90T Δ*ybjX*Δ*virK* more than doubled the production of IL-8 compared to the wild-type strain and significantly increased the released IL-8 with respect to the Δ*virK* mutant (Figure 6A). This result indicates that YbjX influences the extent of cell response activation, a role that emerges only in the absence of VirK. Notably, infection of HEK-293 with any deletion mutant did not result in a significant change in the amount of secreted IL-8 (Figure 6B). Since HEK-293 cells express neither TLR2 nor TLR4 [43], these data suggest that the improved release of IL-8 by Caco-2 in response to the infection with M90T Δ*virK* and Δ*ybjX*Δ*virK* derivatives likely relies on the activation of the TLR signaling pathways.

### Lack of *YbjX* and *VirK* exacerbates infected macrophage cell death and affects the viability of intracellular *Shigella*

**Figure 7.**
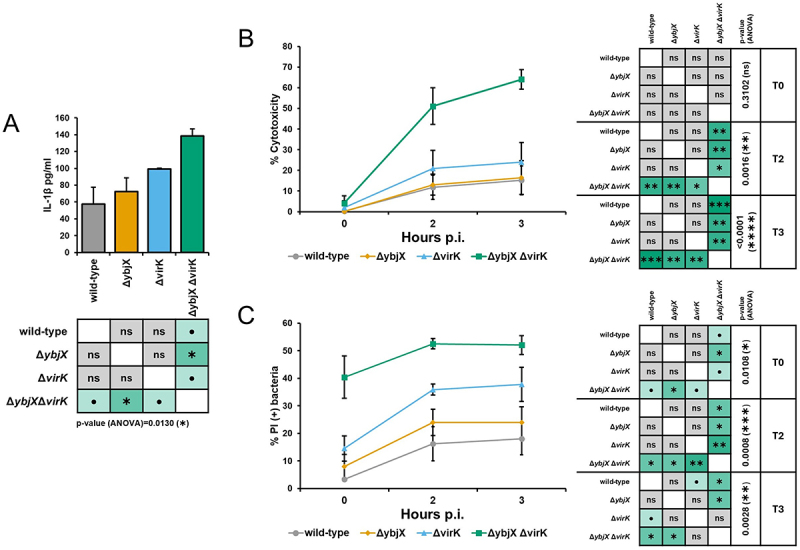
Deletion of *ybjX* and *virK* influences IL-1β release by infected macrophages and affects bacterial intracellular survival. (A) IL-1β released by macrophages differentiated from THP-1 cells was measured by ELISA assay on supernatants recovered from cells infected with M90T and its derivatives 3 hours p.I. (B) THP-1 cytotoxicity was evaluated by quantifying the release of lactate dehydrogenase by infected cells at different time points post-infection, results are expressed as percentages relative to a positive control where all cells were lysed. (C) Viability of intracellular bacteria recovered at different time points p.i. was assessed by DAPI/PI double staining. Results are expressed as the percentage of PI (+) dead bacteria over the whole bacterial population that was stained with DAPI. All infection experiments were repeated three times and at least three wells were run for each sample, when applicable. Statistical significance was determined using a one-way ANOVA test with Tamhane’s T2 post-hoc test. ●0.1≥*p* > 0.05; *0.05≥*p* > 0.01; **0.01≥*p* > 0.001; ***0.001≥*p* > 0.0001. Error bars represent SD.

The infection of THP-1-derived macrophages induces, although to a lesser extent compared to the infected Caco-2 epithelial cells, a rise in both *ybjX* and *virK* transcripts (Figure 4A). Building on what was observed in epithelial cells regarding the modulation of the innate response, we hypothesized that the lack of these functions could influence the release of pro-inflammatory cytokines by infected macrophages too. To test this hypothesis, we performed infections of THP-1-derived macrophages using the M90T wild-type strain and the Δ*ybjX*, Δ*virK* or Δ*ybjX*Δ*virK* derivatives and analyzed the concentration of IL-1β secreted by infected cells 2 hours p.i. The infection with M90TΔ*ybjX* did not significantly influence the release of IL-1β, while M90T *ΔvirK* caused a significant increase in the concentration of IL-1β in the supernatant (Figure 7A). Interestingly, infection with the strain lacking both *ybjX* and *virK* genes further enhanced the release of IL-1β, supporting once again a role for YbjX. It is well acknowledged that the release of IL-1β by macrophages upon *Shigella* invasion results from the activation of inflammatory caspases, which ultimately leads to pyroptotic cell death [1,44]. Accordingly, we explored whether any relationship exists between increased IL-1β processing and macrophage cell death. As shown in Figure 7B, cell death of THP-1-derived macrophages infected with the double deletion mutant increased by around five times at 2 and 3 hours p.i. with respect to the wild-type strain. The single M90T deletion mutants had no significant effect on macrophage cell death compared to the wild-type strain. Finally, we investigated whether the lack of YbjX and/or VirK and the high cytotoxicity observed in macrophages impact intracellular bacteria. We assessed the survival ability of M90T and its derivatives inside THP-1-derived macrophages by DAPI/PI double staining. As shown in Figure 7C, deletion of both *ybjX* and *virK* genes profoundly affected bacterial viability as soon as entry into host cells. Indeed, at T0, corresponding to 30 minutes after the addition of bacteria, 40% of the intracellular *Shigella* derivatives were found PI positive, meaning that the dead Δ*ybjX*Δ*virK* bacteria were 10 times higher than the wild-type. Even the mere absence of VirK negatively impacts intracellular survival, although to a lesser extent. A significant decrease of viable bacteria was visible at 2 hours p.i, with an increment of dead Δ*virK* bacteria by more than two times with respect to the wild-type *Shigella*. On the contrary, the lack of YbjX alone did not significantly influence the viability phenotype. However, deletion of *ybjX* in a *virK* defective background becomes highly relevant for the survival of intracellular bacteria, revealing a rescue-like function of YbjX when VirK is absent.

## Discussion

In this work, we characterized the genetic loci of the *S. flexneri* YbjX and VirK proteins and we evaluated their relevance in the defense against antimicrobial compounds and the surveillance of the host immune system. Our results indicate that both YbjX and VirK play a role in the maintenance of OM permeability, possibly acting as modifiers of molecules involved in OM biogenesis/remodeling. Furthermore, we provide evidence demonstrating that their activity is pivotal for *Shigella* infection of macrophages and epithelial cells, as their absence leads to an abnormal host inflammatory response and severely affects *Shigella* viability inside macrophages. The *ybjX* gene was first described in *Salmonella* as a suppressor of the *msbB* mutation [10]. Subsequently, the *Salmonella* YbjX protein has been reported to share 35% identity with *Salmonella* VirK and 36% with *Shigella* VirK, whose gene was the first to be identified. *Shigella virK* is one of the genes gained through the acquisition of the large virulence plasmid (pINV) that defines the *Shigella*/EIEC pathogroup, and it is part of the *shf-rfbU-virK-msbB2* operon. VirK has been known for at least three decades and, though previous findings pointed to a role in the regulation of actin-based motility required for the colonization of the colonic epithelium [16,17] it is still unclear how this regulation is achieved and whether VirK is involved in other functions. In *Salmonella* and *Campylobacter jejuni* the deletion of *virK* homologues is associated with membrane instability and decreased virulence [14,18]. Regarding YbjX, although the coding gene is conserved across *Enterobacteriaceae*, it gained more attention only after the observation that it displays a high similarity with VirK. The *ybjX* gene belongs to the set of genes lacking experimental evidence of function. Approximately 35% of *E. coli* genes are included in this so-called y-ome, and the percentage rises for less-studied bacterial species [9]. Few data obtained in *Salmonella* and avian pathogenic *E. coli* (APEC) suggest a role in resistance to antimicrobial peptides and virulence [11–15]. The *ybjX* gene is well conserved in the *Shigella* chromosome and is transcribed from a promoter partially overlapping the divergent *macAB* promoter (Figure 1 and S1). Translation of YbjX can alternatively start from two close ATG codons (Figure 1D). Interestingly, the presence of two methionine residues located 19 and 25 bases downstream of the transcriptional start site appears to be a conserved feature among *ybjX* genes from diverse species. The expression of the *virK* gene is known to be positively regulated by the PhoPQ TCS [33]. This also applies to the *ybjX* gene, as its expression is silenced in the absence of either PhoPQ components or in the presence of Mg^2 +^ (Figure 2). Most of the PhoP-regulated genes are involved in virulence and the homeostasis of the cell envelope, its synthesis and modification [40]. Indeed, we found that both proteins are involved in the maintenance of a proper OM permeability, as suggested by the increased sensitivity of *Shigella* derivatives to novobiocin and polymyxin B. The involvement of VirK in the maintenance of the envelope integrity can be inferred also by the genetic context of the *virK* gene. Indeed, the other genes of the *shf-rfbU-virK-msbB2* operon have been studied for their roles in membrane-related processes. For instance, Shf shares a partial amino acid identity (25.6%) with a metal-dependent N-acetylase of *Staphylococcus epidermidis* [45] and is required for biofilm formation by EAEC [46], and the RfbU is predicted to possess a LPS glycosyltransferase activity [47]. Interestingly the *msbB2* gene encodes a myristoyl transferase that catalyses the addition of a myristate group to penta-acyl lipid A and its paralogue *msbB1* is found on the chromosome [33,48]. Concerning YbjX, as mentioned above, it was first identified in *Salmonella* among suppressors of *msbB* mutant unable to myristoylate lipid A within LPS [10]. Our analysis suggests that YbjX and VirK share the same architecture as many proteins of the Gcn5-related N-acetyltransferase (GNAT) superfamily, which includes enzymes that catalyze the transfer of an acetyl group from acyl-CoA to a recipient molecule. We assessed the localization of YbjX and VirK in the cytoplasm (Figure 3F), hence excluding a structural role as membrane or membrane-associated proteins. We hypothesize that YbjX and VirK may act as post-translational regulators of other proteins directly involved in the integrity and/or the synthesis of the outer membrane and, probably, of LPS. Infection with the M90T derivatives elicits an increased release of proinflammatory cytokines by both macrophages and epithelial cells. Noteworthy, the lack of YbjX and VirK in infecting *Shigella* enhances IL-8 secretion only in those cells proficient for sensing LPS, such as Caco-2, while does not affect the response of HEK-293 cells that are known to be defective for the expression of some TLRs, including TLR4 [43], as well as for inflammatory caspases known to sense intracellular LPS [49]. Altogether these findings point to a relevant role of YbjX and VirK in the OM biogenesis, particularly in the LPS synthesis/modification, allowing *Shigella* to better modulate the host immune response. As discussed above, YbjX and VirK display a high structural similarity that, however, does not translate into complete functional redundancy. Generally, the lack of YbjX did not have an impact on the phenotype of *Shigella*, unlike the absence of VirK, which affected almost every parameter that was taken into consideration. Interestingly, the deletion of the *ybjX* gene alone was phenotypically silent unless coupled with the deletion of *virK*. In that case, a more dramatic phenotype emerged. The *S. flexneri* M90T Δ*ybjX*Δ*virK* strain displayed extreme sensitivity to novobiocin (Figure 3B) and the cationic antimicrobial peptide polymyxin B (Figure 3C). Moreover, the Δ*ybjX*Δ*virK* mutant induced the most abundant release of the proinflammatory cytokines IL-8 (Figure 6A) and IL-1β (Figure 7A) by infected Caco-2 epithelial cells and THP-1-derived macrophages, respectively. Additionally, removing both genes resulted in a severe cytotoxic effect on infected macrophages (Figure 7B) and greatly affected bacterial viability in the macrophage intracellular environment (Figure 7C). These observations suggest that the presence of at least one of the two proteins is required to ensure bacterial survival in various conditions and indicate that the acquisition of VirK aimed at strengthening YbjX functions. The *ybjX* gene is strongly conserved in *E. coli*, its pathogenic derivatives, and in other bacterial species with different lifestyles, suggesting an ancient origin and an important protein-associated function. Genome shrinking is common among bacteria that evolved from commensals to pathogens, and *Shigella* is not an exception. *Shigella’s* extensive gene decay in the pathoadaptation process concerns many genes whose functions were useless or detrimental to the infection [50]. However, *ybjX* is maintained in the chromosome of all *Shigella* spp. and we demonstrated that it plays a relevant role during the infection process of the bacterium, ensuring the OM integrity. An additional clue of the relevance of YbjX for the new intracellular lifestyle evolved by *Shigella* is that its function has been further reinforced by the acquisition of *virK* along with the virulence plasmid [6]. As shown above, VirK is essential for resistance to antimicrobial peptides and for escaping the host immune response. These features are crucial in an intracellular pathogen that needs to hide from the host immune system in the early stages of the infection and resist to the antimicrobial action of many host-produced compounds. Remarkably, the same or a similar strategy has been adopted by other pathogens with very different infection mechanisms, suggesting that the acquisition of *virK* is a convergent evolutionary pattern. Among pathogenic *E. coli*, an intriguing case is the one of EAEC 042, which, in addition to *ybjX*, possesses a chromosomal copy of the *virK* gene and a plasmid-encoded copy on the pAA plasmid [18,19,51]. Outside the *Escherichia* genus, both *ybjX* and *virK* can be found on the chromosome of *S. enterica* [14,15]. Two variants of YbjX/VirK proteins were identified during a transcriptome analysis of genes upregulated in *Enterobacter hormaechei* during exposure to the cationic antimicrobials colistin and CSA-13 [52]. Additionally, *C. jejuni* VirK is among the very few virulence factors of this pathogen that have homologs in other bacterial species [20], further emphasizing that the acquisition of this protein may represent a pathogenicity signature. Our work is the first study in which the role of YbjX and VirK in *Shigella* is investigated, considering not only the roles of the individual proteins but also their combined action. Our findings suggest that both proteins are involved in the maintenance of OM integrity, crucial for *Shigella* to resist antimicrobial peptides and to evade the host response. The data presented underline the relevance of YbjX and VirK during the invasion of the colonic epithelium, and, more broadly, highlight the evolutionary advantage conferred by the acquisition of VirK .

## Supplementary Material

REVISED Fig S2.JPG

Supplementary file Statistical analysis.docx

Clean Copy of Supplementary Material - QVIR-2025-0088.R2.docx

REVISED Fig S1.JPG

REVISED Fig S3.JPG

## Data Availability

The data that support the findings of this study are openly available in Mendeley Data at http://doi.org/10.17632/x7ctsyzhjs.3 Reference [53]: Coluccia, Marco (2025), “Conserved and acquired: decoding YbjX and VirK in the pathogenicity of Shigella flexneri,” Mendeley Data, V3, doi: 10.17632/x7ctsyzhjs.3
