# Supplementary figures and images for: Conserved and acquired: Decoding YbjX and VirK in the pathogenicity of Shigella flexneri
Source: Virulence. 2025 Oct 27;16(1):2571677. doi: 10.1080/21505594.2025.2571677 (PMC12562796; doi:10.1080/21505594.2025.2571677)

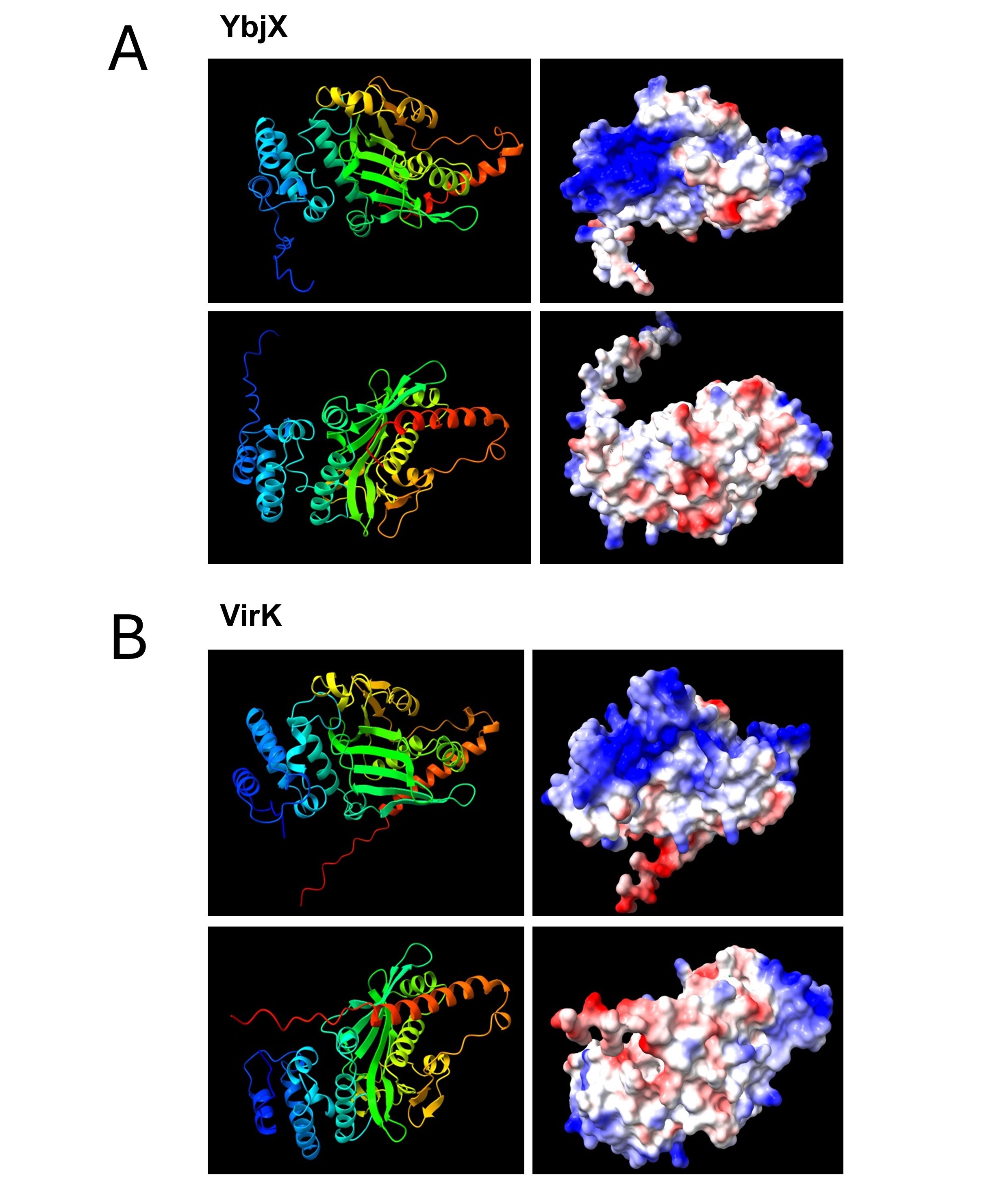

Supplement: REVISED Fig S2.JPG [file KVIR_A_2571677_SM4292.jpg]

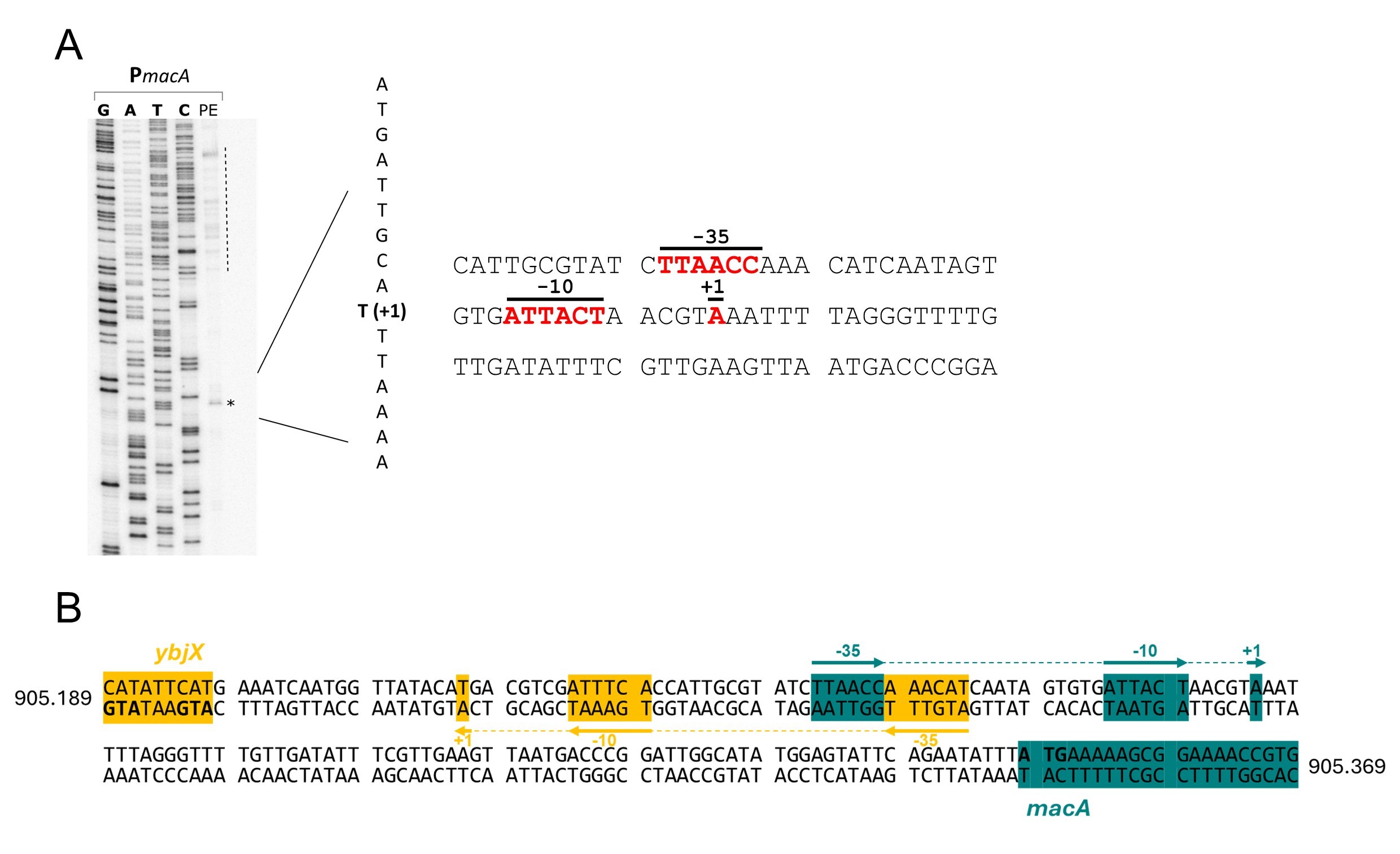

Supplement: REVISED Fig S1.JPG [file KVIR_A_2571677_SM4289.jpg]

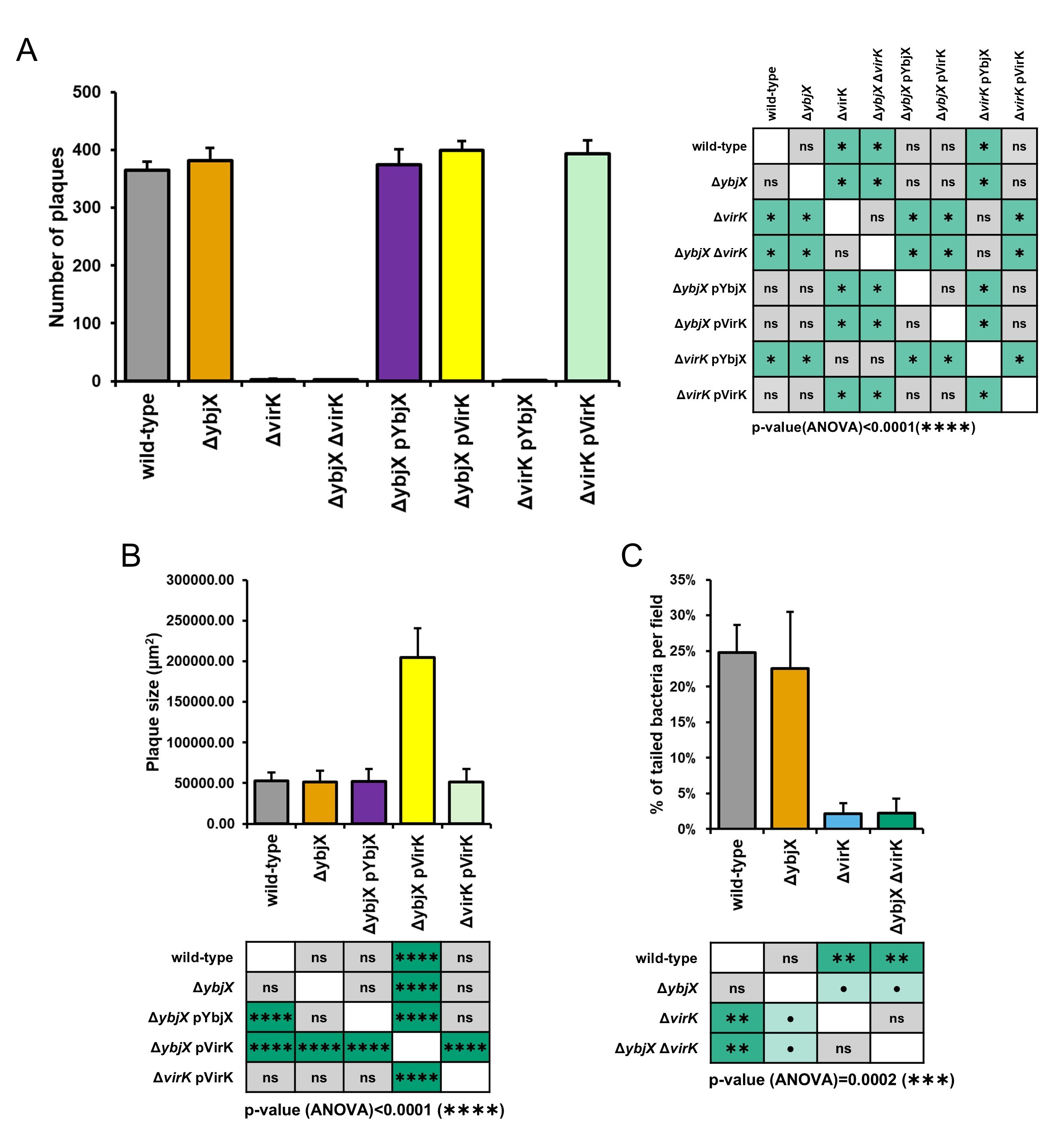

Supplement: REVISED Fig S3.JPG [file KVIR_A_2571677_SM4288.jpg]
