# Supplementary material for: Conserved and acquired: Decoding YbjX and VirK in the pathogenicity of Shigella flexneri
Source: Virulence. 2025 Oct 27;16(1):2571677. doi: 10.1080/21505594.2025.2571677 (PMC12562796; doi:10.1080/21505594.2025.2571677)
Supplement: Supplementary file Statistical analysis.docx [file KVIR_A_2571677_SM4291.docx]

**Fig 1C**

p-value (t-test)=0.0002 (***)

**Fig 1D**

p-value (Welch’s ANOVA)=0.0001 (***)

|  | wild-type | M13L | M15L | M13L M15L |
| --- | --- | --- | --- | --- |
| wild-type |  | 0.5484 | >0.9999 | 0.0168 |
| M13L | 0.5484 |  | 0.7177 | 0.0062 |
| M15L | >0.9999 | 0.7177 |  | 0.0323 |
| M13L M15L | 0.0168 | 0.0062 | 0.0323 |  |

**Fig 2A**

p-value (Welch’s ANOVA)<0.0001 (****)

|  |  | 1 mM MgSO_4_ | | | 0.1 mM MgSO_4_ | | |
| --- | --- | --- | --- | --- | --- | --- | --- |
|  |  | wild-type | Δ*phoP* | Δ*phoQ* | wild-type | Δ*phoP* | Δ*phoQ* |
| 1 mM MgSO_4_ | wild-type |  | 0.0004 | 0.0021 | 0.0162 | 0.0036 | 0.0026 |
|  | Δ*phoP* | 0.0004 |  | 0.2689 | 0.0006 | >0.9999 | 0.2589 |
|  | Δ*phoQ* | 0.0021 | 0.2689 |  | 0.0005 | 0.5864 | >0.9999 |
| 0.1 mM MgSO_4_ | wild-type | 0.0162 | 0.0006 | 0.0005 |  | 0.0003 | 0.0003 |
|  | Δ*phoP* | 0.0036 | >0.9999 | 0.5864 | 0.0003 |  | >0.9999 |
|  | Δ*phoQ* | 0.0026 | 0.2589 | >0.9999 | 0.0003 | >0.9999 |  |

**Fig 2C**

p-value (t-test)=0.0005 (***)

**Fig 4A**

For *ybjX:* p-value (Welch’s ANOVA)<0.0001 (****)

|  | T0 | T2 | T3 |
| --- | --- | --- | --- |
| Ct | 0.0003 | <0.0001 | <0.0001 |

For *virK*: p-value (Welch’s ANOVA)<0.0001 (****)

|  | T0 | T2 | T3 |
| --- | --- | --- | --- |
| Ct | 0.0028 | <0.0001 | <0.0001 |

**Fig 4B**

For *ybjX*: p-value (Welch’s ANOVA)=0.0011 (**)

|  | T0 | T2 | T4 |
| --- | --- | --- | --- |
| Ct | 0.0113 | 0.0136 | 0.0259 |

For *virK*: p-value (Welch’s ANOVA)<0.0001 (****)

|  | T0 | T2 | T3 |
| --- | --- | --- | --- |
| Ct | 0.0484 | 0.0006 | 0.0620 |

**Fig 6A**

p-value (Welch’s ANOVA) <0.0001 (****)

|  | wild-type | Δ*ybjX* | Δ*virK* | Δ*ybjX*Δ*virK* |
| --- | --- | --- | --- | --- |
| wild-type |  | 0.0797 | 0.0661 | 0.0031 |
| Δ*ybjX* | 0.0797 |  | 0.0222 | 0.0023 |
| Δ*virK* | 0.0661 | 0.0222 |  | 0.0073 |
| Δ*ybjX*Δ*virK* | 0.0031 | 0.0023 | 0.0073 |  |

**Fig 6B**

p-value (Welch’s ANOVA)=0.2610 (ns)

**Fig 7A**

p-value (Welch’s ANOVA)=0.0130 (*)

|  | wild-type | Δ*ybjX* | Δ*virK* | Δ*ybjX*Δ*virK* |
| --- | --- | --- | --- | --- |
| wild-type |  | 0.9441 | 0.3505 | 0.0609 |
| Δ*ybjX* | 0.9441 |  | 0.4879 | 0.0498 |
| Δ*virK* | 0.3505 | 0.4879 |  | 0.0881 |
| Δ*ybjX*Δ*virK* | 0.0609 | 0.0498 | 0.0881 |  |

**Fig 7B**

T0

p-value (Welch’s ANOVA)=0.3102 (ns)

|  | wild-type | Δ*ybjX* | Δ*virK* | Δ*ybjX*Δ*virK* |
| --- | --- | --- | --- | --- |
| wild-type |  | >0.9999 | 0.9317 | 0.5610 |
| Δ*ybjX* | >0.9999 |  | 0.9319 | 0.5607 |
| Δ*virK* | 0.9317 | 0.9319 |  | 0.9628 |
| Δ*ybjX*Δ*virK* | 0.5610 | 0.5607 | 0.9628 |  |

T2

p-value (Welch’s ANOVA)=0.0016 (**)

|  | wild-type | Δ*ybjX* | Δ*virK* | Δ*ybjX*Δ*virK* |
| --- | --- | --- | --- | --- |
| wild-type |  | 0.9997 | 0.5870 | 0.0036 |
| Δ*ybjX* | 0.997 |  | 0.6954 | 0.0051 |
| Δ*virK* | 0.5870 | 0.6954 |  | 0.0174 |
| Δ*ybjX*Δ*virK* | 0.0036 | 0.0051 | 0.0174 |  |

T3

p-value (Welch’s ANOVA)<0.0001(ns)

|  | wild-type | Δ*ybjX* | Δ*virK* | Δ*ybjX*Δ*virK* |
| --- | --- | --- | --- | --- |
| wild-type |  | >0.9999 | 0.7105 | 0.0004 |
| Δ*ybjX* | >0.9999 |  | 0.8515 | 0.0011 |
| Δ*virK* | 0.7105 | 0.8515 |  | 0.0065 |
| Δ*ybjX*Δ*virK* | 0.0004 | 0.0011 | 0.0065 |  |

**Fig 7C**

T0

p-value (Welch’s ANOVA)=0.0108(*)

|  | wild-type | Δ*ybjX* | Δ*virK* | Δ*ybjX*Δ*virK* |
| --- | --- | --- | --- | --- |
| wild-type |  | 0.7508 | 0.2651 | 0.0795 |
| Δ*ybjX* | 0.7508 |  | 0.6176 | 0.0389 |
| Δ*virK* | 0.2651 | 0.6176 |  | 0.0720 |
| Δ*ybjX*Δ*virK* | 0.0795 | 0.0389 | 0.0720 |  |

T2

p-value (Welch’s ANOVA)=0.0008(***)

|  | wild-type | Δ*ybjX* | Δ*virK* | Δ*ybjX*Δ*virK* |
| --- | --- | --- | --- | --- |
| wild-type |  | 0.6660 | 0.1304 | 0.0352 |
| Δ*ybjX* | 0.6660 |  | 0.1920 | 0.0255 |
| Δ*virK* | 0.1304 | 0.1920 |  | 0.0027 |
| Δ*ybjX*Δ*virK* | 0.0352 | 0.0255 | 0.0027 |  |

T3

p-value (Welch’s ANOVA)=0.0028 (**)

|  | wild-type | Δ*ybjX* | Δ*virK* | Δ*ybjX*Δ*virK* |
| --- | --- | --- | --- | --- |
| wild-type |  | 0.8596 | 0.0715 | 0.0121 |
| Δ*ybjX* | 0.8596 |  | 0.1885 | 0.0202 |
| Δ*virK* | 0.0715 | 0.1885 |  | 0.2175 |
| Δ*ybjX*Δ*virK* | 0.0121 | 0.0202 | 0.2175 |  |

**Fig S3A**

p-value (Welch’s ANOVA)=<0.0001 (****)

|  | wild-type | Δ*ybjX* | Δ*virK* | Δ*ybjX*Δ*virK* | Δ*ybjX* pYbjX | Δ*ybjX* pVirK | Δ*virK* pYbjX | Δ*virK* pVirK |
| --- | --- | --- | --- | --- | --- | --- | --- | --- |
| wild-type |  | >0.9999 | 0.0141 | 0.0150 | >0.9999 | 0.8060 | 0.0155 | 0.9958 |
| Δ*ybjX* | >0.9999 |  | 0.0313 | 0.0320 | >0.9999 | >0.9999 | 0.0323 | >0.9999 |
| Δ*virK* | 0.0141 | 0.0313 |  | >0.9999 | 0.0488 | 0.0146 | >0.9999 | 0.0330 |
| Δ*ybjX*Δ*virK* | 0.0150 | 0.0320 | >0.9999 |  | 0.0495 | 0.0154 | >0.9999 | 0.0336 |
| Δ*ybjX* pYbjX | >0.9999 | >0.9999 | 0.0488 | 0.0495 |  | 0.9998 | 0.0497 | >0.9999 |
| Δ*ybjX* pVirK | 0.8060 | >0.9999 | 0.0146 | 0.0154 | 0.9998 |  | 0.0158 | >0.9999 |
| Δ*virK* pYbjX | 0.0155 | 0.0323 | >0.9999 | >0.9999 | 0.0497 | 0.0158 |  | 0.0339 |
| Δ*virK* pVirK | 0.9958 | >0.9999 | 0.0330 | 0.0336 | >0.9999 | >0.9999 | 0.339 |  |

**Fig S3B**

p-value (Welch’s ANOVA)=<0.0001 (****)

|  | wild-type | Δ*ybjX* | Δ*ybjX* pYbjX | Δ*ybjX* pVirK | Δ*virK* pYbjX |
| --- | --- | --- | --- | --- | --- |
| wild-type |  | >0.9999 | >0.9999 | <0.0001 | >0.9999 |
| Δ*ybjX* | >0.9999 |  | >0.9999 | <0.0001 | >0.9999 |
| Δ*ybjX* pYbjX | >0.9999 | >0.9999 |  | <0.0001 | >0.9999 |
| Δ*ybjX* pVirK | <0.0001 | <0.0001 | <0.0001 |  | <0.0001 |
| Δ*virK* pVirK | >0.9999 | >0.9999 | >0.9999 | <0.0001 |  |

**Fig S3C**

p-value (Welch’s ANOVA)=0.0002 (***)

|  | wild-type | Δ*ybjX* | Δ*virK* | Δ*ybjX*Δ*virK* |
| --- | --- | --- | --- | --- |
| wild-type |  | 0.9978 | 0.0027 | 0.0015 |
| Δ*ybjX* | 0.9978 |  | 0.0750 | 0.0708 |
| Δ*virK* | 0.0027 | 0.0750 |  | >0.9999 |
| Δ*ybjX*Δ*virK* | 0.0015 | 0.0708 | >0.9999 |  |
