# Supplementary material for: Conserved and acquired: Decoding YbjX and VirK in the pathogenicity of Shigella flexneri
Source: Virulence. 2025 Oct 27;16(1):2571677. doi: 10.1080/21505594.2025.2571677 (PMC12562796; doi:10.1080/21505594.2025.2571677)
Supplement: Clean Copy of Supplementary Material - QVIR-2025-0088.R2.docx [file KVIR_A_2571677_SM4290.docx]

**Supporting information**

**Fig S1. Identification of the *macAB* promoter region**.
A) Identification of the *macA* promoter. The 5' end of the *macA* transcript was identified through primer extension analysis. On the right, a summary of the relevant features within the nucleotide sequence of the *macA* promoter region is presented. The transcriptional start site (+1) and the -10 and -35 regions are shown in red. B) Intergenic region between *ybjX* (in yellow) and *macA* (in blue). The promoters are represented with dashed lines and the arrows indicate the -35, -10 and the +1 regions.

**Fig S2. Alphafold-predicted YbjX (A) and VirK (B) structures showing the charge distribution on the protein surface.**The right panels show the charge distribution on the protein surface (red: negative charge; blue: positive charge). For both YbjX and VirK, in the lower panels the structures are rotated by 180° to show the opposite face of the protein. The charge distribution was calculated with Chimera X.

**Fig S3. Virk, but not YbjX, is involved in cell-to-cell spreading.**A) M90T strains lacking VirK are unable to form plaques on epithelial cells and *virK* deletion can’t be complemented by the overexpression of YbjX (Δ*virK* pYbjX). B)Overexpression of VirK increases plaque size produced by M90T Δ*ybjX*. For plaque assays, a confluent layer of Caco-2 cells was infected with *S. flexneri* M90T wild-type and its derivatives at MOI 10^-3^. Plaque size was measured 72 hours p.i.. C) The deletion of *virK* disrupts the ability of *Shigella* to polymerise actin tails during the infection of epithelial cells. The percentage of tailed bacteria was calculated in n=4 60x obj fields for each strain infecting epithelial cells, 4 hours post-infection.
Statistical significance was determined using a one-way ANOVA test with Tamhane’s T2 post-hoc test. ●0.1≥p>0.05; ** 0.01≥p>0.001; **** p≤0.0001. Error bars represent SD.

**Table S1. Strains and plasmids used in this study.**

| **Strain** | **Relevant characteristics** | **Source/reference** |
| --- | --- | --- |
| M90T | M90T *S. flexneri* 5a | Pasteur Institute, Paris [7] |
| M90T Δ*ybjX* | M90T derivative defective in *ybjX* gene, Km^R^ | This study |
| M90T Δ*ybjX* Km^S^ | M90T derivative defective in *ybjX* gene, Km^S^ | This study |
| M90T Δ*virK* | M90T derivative defective in *virK* gene, Cm^R^ | This study |
| M90T Δ*virK* Cm^S^ | M90T derivative defective in *virK* gene, Cm^S^ | This study |
| M90T Δ*ybjX* Δ*virK* | M90T derivative defective in *ybjX* and *virK* genes, Km^R^ Cm^R^ | This study |
| M90T Δ*phoP* | M90T derivative defective in *phoP* gene, Km^R^ | This study |
| M90T Δ*phoQ* | M90T derivative defective in *phoQ* gene, Km^R^ | This study |
| M90T YbjX-His | M90T with YbjX-tagged (His-tag) protein, Km^R^ | This study |
| M90T VirK-His | M90T with VirK-tagged (His-tag) protein, Km^R^ | This study |
| DH10b | F– *mcrA* Δ(*mrr-hsdRMS-mcrBC*) φ80*lacZ*ΔM15 Δ*lacX*74 *recA*1 *endA*1 *araD*139 Δ (*ara-leu*)7697 *galU galK* λ– *rpsL*(Str^R^) *nupG* | EC0113, Thermo Fisher Scientific [54] |
| **Plasmid** | **Relevant characteristics** | **Source/reference** |
| pRU1097 | GFP reporter plasmid, Gm^R^. | [55] |
| pRU*macA* | pRU1097-derived vector carrying the regulatory region of the *macA* gene in *BamHI* site, Gm^R^ | This study |
| pRU*ybjX* | pRU1097-derived vector carrying the regulatory region of the *ybjX* gene in *BamHI* site, Gm^R^ | This study |
| pRU*ybjX-*MUT | pRU*ybjX-*derived vector carrying site-directed mutations in the *ybjX* gene -10 promoter sequence, Ap^R^ | This study |
| pRU*virK* | pRU1097-derived vector carrying the regulatory region of the *shf-rfbU-virK-msbB2* operon in *BamHI* site, Gm^R^ | This study |
| pSUB11 | Plasmid carrying a kanamycin resistance cassette, template for PCR, Km^R^ | [56] |
| pACYC184 | Cloning vector carrying the p15A origin of replication, Cm^R^, Tc^R^. | [57] |
| pYbjX-His | pACYC184-derived vector carrying the *ybjX*-tagged gene (His tag) in *BamHI* site*,* Km^R^ | This study |
| pYbjX-His-M13L | pYbjX-His-derived vector carrying M13L mutation of the *ybjX* gene, Km^R^ | This study |
| pYbjX-His-M15L | pYbjX-His-derived vector carrying M15L mutation of the *ybjX* gene, Km^R^ | This study |
| pYbjX-His-M13L M15L | pYbjX-His-derived vector carrying M13L and M15L mutations on the *ybjX* gene, Km^R^ | This study |
| pGIP7 | pACYC184 derivative carrying Ptac promoter, Cm^R^ | [58] |
| pYbjX | pGIP7 derivative carrying the *ybjX* gene in *BamHI* site, Cm^R^ | This study |
| pVirK | pGIP7 derivative carrying the *virK* gene in *BamHI* site, Cm^R^ | This study |
| pKD46 | Red recombinase expression plasmid, Ap^R^ | [22] |
| pKD3 | Template plasmid carrying a kanamycin resistance gene flanked by Flp recognition target sequences, Cm^R^, Ap^R^ | [22] |
| pKD4 | Template plasmid carrying a kanamycin resistance gene flanked by Flp recognition target sequences, Km^R^, Ap^R^ | [22] |
| pCP20 | Temperature-sensitive replicon carrying the yeast Flp recombinase gene, Ap^R^ | [22] |

**Table S2. Oligonucleotides used in this study.**

| **Chromosomal deletion** |  |
| --- | --- |
| **Name** | **5’-3’ sequence** |
| ybjX-F | TGCGTCTGCATCGCCCTTACCTTGCCGCGAATCTTAGCCGATATGAATATCCTCCTTAGT |
| ybjX-R | ATCGTGGCCATTTGTGGCTGAATAGCGTCGAGCATCTCATTGTGTAGGCTGGAGCTGCTTC |
| virK-F | CAATATGTTTTCTGTAAGTAACTTATCATTTATCGGTTTCTGTGTAGGCTGGAGCTGCTTC |
| virK-R | TTAAGTCCTGATGTTCTGAGTTTACAGGATATGCATCCAAATATGAATATCCTCCTTAGT |
| phoP-F | GCTGGTCATCAGGTCGATGACGCAGAAGATGCCAAAGAAGTGTGTAGGCTGGAGCTGCTTC |
| phoP-R | AATTCGAACAGGTAGCCCTGGCCGCGAACGGTGGTAATGAATATGAATATCCTCCTTAGT |
| phoQ-F | CCCGCTCTCGCTGCGGGTACGTTTTCTGTTGGCAACGGCATGTGTAGGCTGGAGCTGCTTC |
| phoQ-R | TTTCGGCGCAGAATGCTGGCGACCAAAAATCACCTCCATCATATGAATATCCTCCTTAGT |
| **Protein tagging** |  |
| **Name** | **5’-3’ sequence** |
| ybjX-His-A | GCAATGGTGAAATCGACGTCATG |
| ybjX-His-B | AGCAGCTCCAGCCTACATTACTAGTGATGGTGATGGTGATGACCGCGAAACATCGTGGCCA |
| Kan-His-C | TAATGTAGGCTGGAGCTGCT |
| ybjX-His-D | GGACTCCGTACCAACGCTGCTGAAAAAAATGTTCTCCATATGAATATCCTCCTTAG |
| virK-His-A | TTATCAGATGGCTCGCCAGG |
| virK-His-B | AGCAGCTCCAGCCTACATTACTAGTGATGGTGATGGTGATGATTTAAGTCCTGATGTTCTG |
| virK-His-D | TTTAGTGCGAATGCCCCTACCAAAATCATCATTAATACATATGAATATCCTCCTTAG |
| **Plasmid construction** |  |
| **Name** | **5’-3’ sequence** |
| pRUybjX-BamHI-R | NNNGGATCCATTGAGATTAGGCCAGTGGG |
| pRUybjX-BamHI-F | NNNGGATCCACCGACAGCGTTTTCAAC |
| pRUybjX-MUT-F | GATACGCAATGGCGAATTCGACGTCATGTA |
| pRUybjX-MUT-R | TACATGACGTCGAATTCGCCATTGCGTATC |
| pYbjX-BamHI-F | NNNGGATCCATGGTGAAATCGACGTCATG |
| pYbjX-BamHI-R | NNNGGATCCCATTGAAGTGGCAATGGAG |
| pVirK-BamHI-F | NNNGGATCCAATATGTTTTCTGTAAGTAAC |
| pVirK-BamHI-R | NNNGGATCCTTAATTTAAGTCCTGATGTTC |
| pYbjX-His-BamHI-R | NNNGGATCCTGTTCTCCATATGAATATCCTCC |
| pYbjX-M13L-F | ATAACCATTGATTTCCTGAATATGTCGCAGCTA |
| pYbjX-M13L-R | TAGCTGCGACATATTCAGGAAATCAATGGTTAT |
| pYbjX-M15L-F | ATTGATTTCATGAATCTGTCGCAGCTAACTGAA |
| pYbjX-M15L-R | TTCAGTTAGCTGCGACAGATTCATGAAATCAAT |
| pYbjX-M13L-M15L-F | ATAACCATTGATTTCCTGAATCTGTCGCAGCTAACTGAA |
| pYbjX-M13L-M15L-R | TTCAGTTAGCTGCGACAGATTCAGGAAATCAATGGTTAT |
| **Primer extension** |  |
| macA-PE3 | GGCGATGACTATCACCAGC |
| ybjX-PE3 | TCAGTTAGCTGCGACATATTC |
| shf-PE2 | GAAAACCCAATAAAGCTGGTGC |
| **qRT-PCR** | |
| **Name** | **5’-3’ sequence** |
| qybjX-F | CCCAAACGCCTCGTGATG |
| qybjX-R | GCAATAATCTGCTCTACCTGCAAA |
| qvirK-F | TCCTTGCAGTGCATTGAGGAT |
| qvirK-R | GCCCCCCTTTATGCAAATAAG |
